# Behavioural characteristics in externalising children with low and elevated risk for dental caries

**DOI:** 10.1007/s40368-016-0256-6

**Published:** 2016-11-09

**Authors:** M. Staberg, J. G. Norén, L. Gahnberg, A. Ghaderi, C. Kadesjö, A. Robertson

**Affiliations:** 1 Department of Pediatric Dentistry, Institute of Odontology, The Sahlgrenska Academy, University of Gothenburg, P.O. Box 450, 405 30 Gothenburg, Sweden; 2 Department of Behavioral and Community Dentistry, Institute of Odontology, The Sahlgrenska Academy, University of Gothenburg, Gothenburg, Sweden; 3 Division of Psychology, Department of Clinical Neuroscience, Karolinska Institutet, Stockholm, Sweden; 4 Gillberg Neuropsychiatry Centre, Institute of Neuroscience and Physiology, The Sahlgrenska Academy, University of Gothenburg, Gothenburg, Sweden

**Keywords:** Child behaviour, Conduct problems, Dental caries, Disruptive behaviour results disorder

## Abstract

**Aim:**

To compare two groups of children with externalising behaviour problems, having low and elevated caries risk, respectively. Those parameters were assessed in relation to behavioural characteristics and family structure, and to compare the caries risk assessment and gender differences in relation to children in general in the Region of Västra Götaland, Sweden.

**Methods:**

Families (228) with children, aged 10-13 years, participating in parent training programmes, were recruited. Parents provided information through questionnaires regarding parental knowledge and monitoring, family warmth and conflict and family structure. Children’s behavioural characteristics, based on the Strength and Difficulties Questionnaire and the Disruptive Behaviour Disorder rating scale, were used as outcome. Data about caries risk assessment were obtained from dental records.

**Results:**

Children in the elevated caries risk group showed higher mean values for conduct problems as well as impulsivity. Parents of the children in the elevated caries risk group reported more parental solicitation and less family conflicts. Children with an elevated caries risk lived more often in households with more than two children and had more often a father from a non-Nordic country.

**Conclusion:**

There were statistically significant more children with an elevated caries risk in the study group compared to children in general in the Region of Västra Götaland, both totally and within gender. Differences were observed with regard to behavioural characteristics in externalising children with an elevated risk for caries. Increased knowledge regarding behavioural characteristics in externalising children is an important parameter to be considered in caries risk assessment.

## Introduction

Children with externalising behaviour problems (EBP) constitute a heterogeneous group of children and refers to behaviour problems manifested in children’s outward behaviour and reflect the child negatively acting on the external environment. EBP includes attention-deficit/hyperactivity disorder (ADHD) problems, as well as disruptive, oppositional, aggressive, and conduct disorder (CD) behaviour (Bloomquist and Schnell 2002). Children who begin to exhibit externalising behaviour in childhood have an increased likelihood of sustained patterns of externalising behaviour across the lifespan and are at increased risk for developing long-term negative outcomes, including antisocial behaviour in adolescence and adulthood (Moffitt and Caspi 2001; Broidy et al. 2003).

The development, maintenance and expression of externalising behaviour problems are also related to parenting, parent–child relationship, and family structure (Bloomquist and Schnell 2002). Parental behaviour risk factors for EBP children are poor supervision and monitoring, lack of parental involvement with the child, as well as harsh and inconsistent discipline (Bloomquist and Schnell 2002; Blazei et al. 2006). Good parental behaviour includes rules, expressed warmth, and knowledge about a child’s whereabouts, which in the long-term influences the child’s comfort in voluntarily sharing information (Wang et al. 2011).

In a review article, conflicting findings were observed in the literature on the association between EBP and dental caries (Rosenberg et al. 2014). Despite improved dental health in Swedish children during decades, it seems plausible to assume that externalising behaviour problems may influence oral health and dental treatment outcome (Arnrup et al. 2003).

In Sweden, all children are assessed for caries risk by their dentist at their regular dental examinations. Caries risk assessment is defined as the probability of an individual patient to develop caries lesions over a certain period of time. An individual caries risk assessment is of importance in order to target prevention resources for children who need it the most (Twetman et al. 2013). Aspects of a child’s behavioural status may be important parameters to be considered in caries risk assessment, as well as for dental treatment and therapy planning.

The aim of the present study was to compare two groups of children with externalising behaviour problems, having low and elevated caries risks, respectively, in relation to behavioural characteristics and family structure and, further, to compare the caries risk assessment and gender differences in relation to children in general in the Region of Västra Götaland (RVG), Sweden.

## Hypothesis

There are more children with externalising behaviour problems having an elevated caries risk, compared to children in general in the Region of Västra Götaland, Sweden. Children with externalising behaviour problems and elevated caries risk are characterised by different behavioural characteristics and family structure, compared to externalising children with low caries risk.

## Materials and methods

### Study group

The study population comprised 228 families with children (10–13 years of age), where the parents experienced the child had externalising behaviour problems. This quantitative cross-sectional dental study is part of a comprehensive study of parent management training (PMT) programmes, examining early intervention for children with externalising behaviour problems. Participants were recruited from different socioeconomic areas in the City of Gothenburg and all data were collected before parents were enrolled in the intervention study.

The families were informed and invited to the study through direct mailings: by letters sent to 13,000 families with children at the targeted ages at all the participating municipalities in Gothenburg, advertisements on bulletin boards, as well as parent meetings at schools. A research assistant contacted the interested parents and conducted a screening interview by telephone, ensuring the families belonged to the target group (e.g. parents with children in conflict with peers, parents or other adults, protesting against demands, often restless, having friends with bad influence or having been involved in vandalism, shoplifting or truancy) (Bjornsdotter 2014).

A total of 796 families who experienced some degree of externalising behaviour problem in their child a willingness to participate in the study. After obtaining written informed consent, all parents were asked to fill out the Strengths and Difficulties Questionnaire (SDQ) (Goodman 1997). Those below a present cutoff point, the criteria for clinically relevant problems (less than 3 points on the conduct problem subscale of the SDQ), and children with autism, obsessive compulsive disorder or on-going psychiatric treatment, were excluded.

**Fig. 1:**
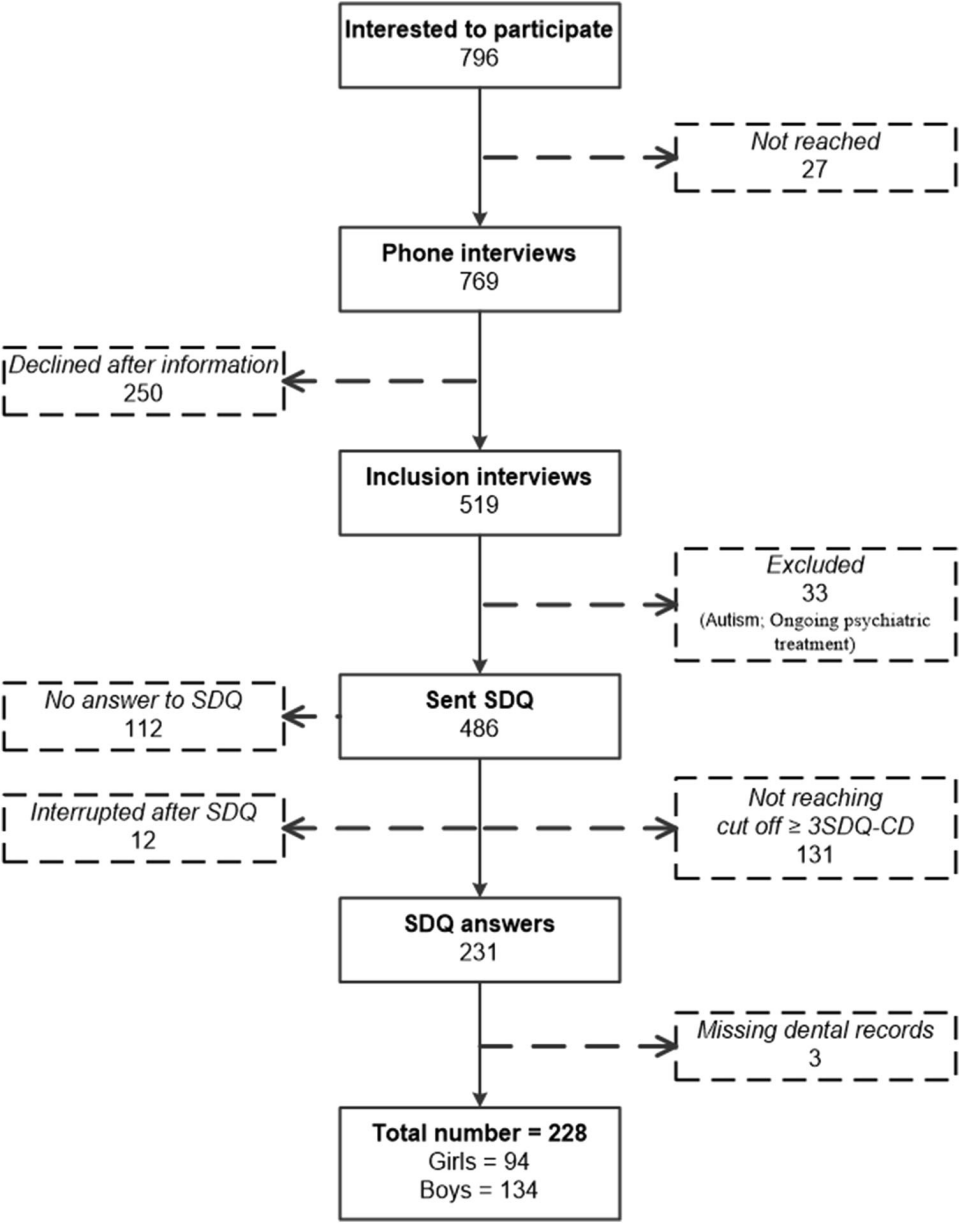
Flow chart describing the recruitment of patients to the study. (SDQ Strengths and Difficulties Questionnaire)

Finally, 231 families entered the study; three children were excluded due to missing dental records, leaving a total of 228 children (134 boys and 94 girls). A flow chart illustrating the recruitment process and those who declined or were excluded is presented in Fig. 1.

### Reference group

Data from the total population of children aged 10-13 years (58,145), in 2013, in the Region of Västra Götaland (RVG), Sweden, served as reference to the study group.

### Normative data

Normative data for SDQ (10–13 years), from 2800 children, were obtained prior to the study from a random selection of families with children at each age (10, 11, 12 and 13 years old), with adequate distribution of both sexes across Sweden using the Swedish Population Address Register (Bjornsdotter et al. 2013).

### Instruments

#### Background information questionnaires

The parents were asked to provide background information through questionnaires about the family structure (parents’ marital status, parents’ native country, number of children in the household).

#### The Strengths and Difficulties Questionnaire (SDQ)

The SDQ (Goodman 1997) is a brief behavioural screening instrument used for children and adolescents with good psychometric properties (Goodman 2001). The SDQ symptom scales contain 25 items divided into five subscales, namely emotional symptoms, conduct problems, hyperactivity–inattention, peer problems, and prosocial behaviour. A 3-point Likert scale is employed to indicate how each attribute applies to the target child (0 = Not true; 1 = Somewhat true; 2 = Certainly true). All subscales, with the exception of Prosocial Behaviour, are summed together to a Total Difficulties score. A high score on the Prosocial Behaviour subscale indicates a strength, while high scores on the other four subscales indicate difficulties.

The parental version of the SDQ for children 4–16 years, used in this study, has been validated for Swedish conditions (Smedje et al. 1999). Due to high skewness and kurtosis on item level, polychoric ordinal alpha was used as a measure of internal consistency instead of Cronbach’s alpha. The internal consistency of the SDQ (polychoric ordinal alpha) ranged between 0.84 and 0.91 (emotional problems: 0.89, hyperactivity–inattention: 0.88, peer problems: 0.84, prosocial behaviour: 0.91, and conduct problems: 0.88).

#### Disruptive behaviour disorder (DBD)

The DBD version used here and responded to by the parents includes 41 items (Bjornsdotter 2014), whereas earlier versions had 45 items (Pelham et al. 1992). The subscales are attention-deficit/hyperactivity disorder (ADHD: 18 items), oppositional defiant disorder (ODD: 8 items) and conduct disorder (CD: 15 items). The items are worded as closely as possible to the DSM (American Psychiatric Association 2000) criteria taking into account the scale format. Each item is rated on a 4-point Likert-type scale (0 = Not at all, 1 = Just a little, 2 = Pretty much, and 3 = Very much). The DBD has shown good psychometric properties (Pelham et al. 1992). The internal consistency (polychoric ordinal alpha) of the subscales of the DBD ranged between 0.94 and 0.99.

#### Family Warmth and Family Conflict (FW/FC)

The questionnaire Family Warmth and Family Conflict consists of five questions regarding warmth and four questions regarding conflict. The items concerning warmth are from the Adult–Child Relationship Scale (Criss and Shaw 2005), which is an adaptation of the School-based Student–Teacher Relationship Scale (Pianta and Nimetz 1991). Internal consistency has previously been shown (Bjornsdotter et al. 2013). The questions on conflict are adapted from the PAL 2 project by the Child and Family Centre, University of Oregon, USA. The Family Warmth subscale is responded to on a 5-point Likert scale from “Definitely not” to “Definitely”. The Family Conflict subscale is responded to on a 7-point Likert scale from “Never” to “More than 7 times during the last month”. A complete list of items can be obtained by contacting the corresponding author. The internal consistency (Cronbach’s alpha) for Family Warmth in the present study was 0.82, and the corresponding value for Family Conflict was 0.72.

#### The Parental Knowledge and Monitoring Scale (PKMS)

The PKMS questionnaire (Stattin and Kerr 2000) consists of two parts: (1) parental knowledge (8 items), providing an overall measurement of parental knowledge (what parents know about their child, the child’s activities and whereabouts), and (2) three subscales measuring different ways of gathering information, including monitoring strategies; parental solicitation (i.e. a way of actively obtaining information/asking questions about the child’s whereabouts) (5 items), parental control (rules and restrictions on the child’s activities) (4 items), and child disclosure (the child’s spontaneously shared information) (5 items). Items are answered on a 5-point Likert scale that ranges from “Almost always” to “Never” or from “Several times a week” to “Never” or from “Very often” to “Almost never” or from “Very good knowledge” to “None or almost no knowledge”.

As a result of subsequent research and investigations of the psychometrics of the PKMS, the first two items on disclosure have been classified into the new Secrecy subscale, and the remaining three questions represent the Child Disclosure subscale. Splitting the Child Disclosure subscale, into Secrecy and Child Disclosure, led to a higher internal consistency for each subscale (Secrecy and Child Disclosure) (Frijns et al. 2010).

The internal consistency (Cronbach’s alpha) of the PKMS subscales in the present study ranged between 0.70 and 0.85 (Parental Knowledge 0.85, Parental Solicitation 0.70, Parental Control 0.81, Child Secrecy 0.80 and Child Disclosure 0.78). A complete list of the items can be obtained by contacting the corresponding author.

#### Caries risk assessment (R2)

All Swedish children are assessed for caries risk at their regular dental recall examinations. Information about caries risk, estimated by the computerised algorithm-based system R2 (Andas and Hakeberg 2014), used by the Public Dental Service in the Region of Västra Götaland (RVG), was obtained from the dental file system. The caries risk clinical assessment is made by child’s regular dentist, according to the regional standardised guidelines by the Region of Västra Götaland. The guidelines can be obtained by contacting the corresponding author (MS).

The caries risk assessment in R2 is conducted in three steps: First, the patient’s current dental caries activity is estimated based on new caries lesions and caries progression in all proximal, buccal and lingual tooth surfaces, including both enamel and dentine caries. Second, modifying factors are recorded such as diet, fluoride usage, oral hygiene, previous caries experience, age and medical risk. Finally, positive and negative factors are weighed by the R2 system to characterise the caries risk as low, intermediate or high. To identify children at risk, data were dichotomised to low and elevated caries risk.

#### Evaluation of caries risk employing inductive analysis

Inductive methods are powerful tools for analyses of, for example, patterns in data sets, and have been used for different applications (Klingberg et al. 1999; Melin et al. 2015). The outcome values in an inductive analysis have to be discrete; however, ingoing attributes can be numerical as well as discrete. Data from the dental records of the patients in the present study were compiled in an Excel spread sheet. As “*attributes”,* the factors “Caries Activity”, “Dietary Habits”, “Oral Hygiene” and “Medical Risk Factors” were set in columns, each having a discrete value “*Low risk*”, “*Intermediate risk*” or “*High risk*” as given in the dental records.

A fifth column was inserted as outcome, representing the caries risk values. As in the main study, intermediate and high caries risk was merged into one group; thus, the two outcome values were “Low Risk” or “Elevated Risk”. The data were imported to the inductive analysis programme XpertRule Analyser (Attar Software, Lancashire, UK). The results are presented in a hierarchic diagram (*knowledge tree*), in which the importance of every attribute in the inductive analysis is specified by its position/level in the knowledge tree. The higher up the tree, the more important for the outcome and, thus, the tree shows how different attributes affect the outcome.

In the analysis, 50% of the examples were randomly selected by the programme for use in the induction of a knowledge tree (*training set*), and the remaining examples were used for verification of the generated rules (*test set*).

### Statistical analyses

The Statistical Package for Social Sciences (SPSS version 21) was used for the statistical analyses. Pearson’s Chi-square test for categorical variables and *t* test for continuous variables were used to analyse family structure and to compare means for the low caries risk group to the elevated caries risk group regarding child behavioural characteristics. Chi-square test was employed for comparing the caries risk assessment between the study group and the reference group. The significant level was set to be *p* < 0.05.

The internal consistencies of the various subscales, where a measure of how closely related a set of items are as a group, were calculated using Cronbach’s alpha, for all instruments. Due to some skewness and/or kurtosis on some items on the SDQ and the DBD, polychoric ordinal alpha (Gadermann et al. 2012) was calculated instead of Cronbach’s alpha when more appropriate. The effect sizes are presented as Cohen’s *d*. A Cohen’s *d* of 0.8 or above was considered a large effect, 0.5 a medium effect, and 0.2 a small effect (Cohen 1988). The Phi coefficient (*φ*) was calculated to estimate the magnitude of the associations of the Chi-square test. A magnitude of 0.5 was considered strong, 0.3 intermediate and 0.1 weak.

### Ethical considerations

The study was approved by the Ethical Committee in Uppsala (dnr 2010/119). All families participating in the project were given written information. Written consent from the participating families was received to partake in the study and to acquire access to their child’s dental records.

## Results

### Study group

The distribution by age was as follows: 59 children (10 years) (25.9%), 46 children (11 years) (20.2%), 45 children (12 years) (19.7%), and 78 children (13 years) (34.2%).

Of the 228 parents answering the questionnaire, 200 were mothers (87.7%) and 28 were fathers (12.3%). There were 66 single parents (53 mothers and 13 fathers). In cases where both parents answered the questionnaires, answers from the parent participating in the intervention/parent training programme were used. All socioeconomic areas in the city of Gothenburg were represented in the study.

### Native country

The parents’ distribution by native country of orifin showed that there were 164 mothers (71.9%) and 135 fathers (59.2%) were Swedish. There were two mothers (0.9%) and nine fathers (3.9%) with an origin from the other Nordic countries. Sixty-two mothers (27.2%) and 84 fathers (36.8%) had an origin from other countries (Table 4).

### Caries risk assessment

There were 153 children in the low caries risk group, 47 children in the intermediate group, and 28 children in the high risk group. The intermediate and high risk groups together formed the elevated caries risk group and consisted of 75 subjects. There were statistically significant more children with an elevated caries risk in the study group, compared with the data found for the children in the reference group in the Region of Västra Götaland, both for the genders (*p* < 0.001) and for the total groups (*p* < 0.001) (Table 1).

**Table 1:**
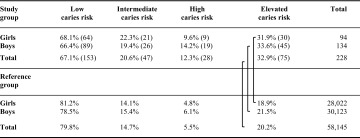
The percentage and number (in brackets) of boys and girls in the low, intermediate and high caries risk groups, and in the elevated caries risk group (combining the intermediate and the high caries risk groups), respectively

The corresponding values are given for the girls, boys and the total number of children in the reference group in the Region of Västra Götaland, Sweden. The reference group having a total number of 58,145 children aged 10–13 years in 2013. The brackets show the statistically significant differences (*p* < 0.001) regarding elevated caries risk between girls, boys and total numbers, respectively, of children with externalising behaviour in the Study group and children in the Reference group

### Evaluation of caries risk employing inductive analysis

**Fig. 2:**
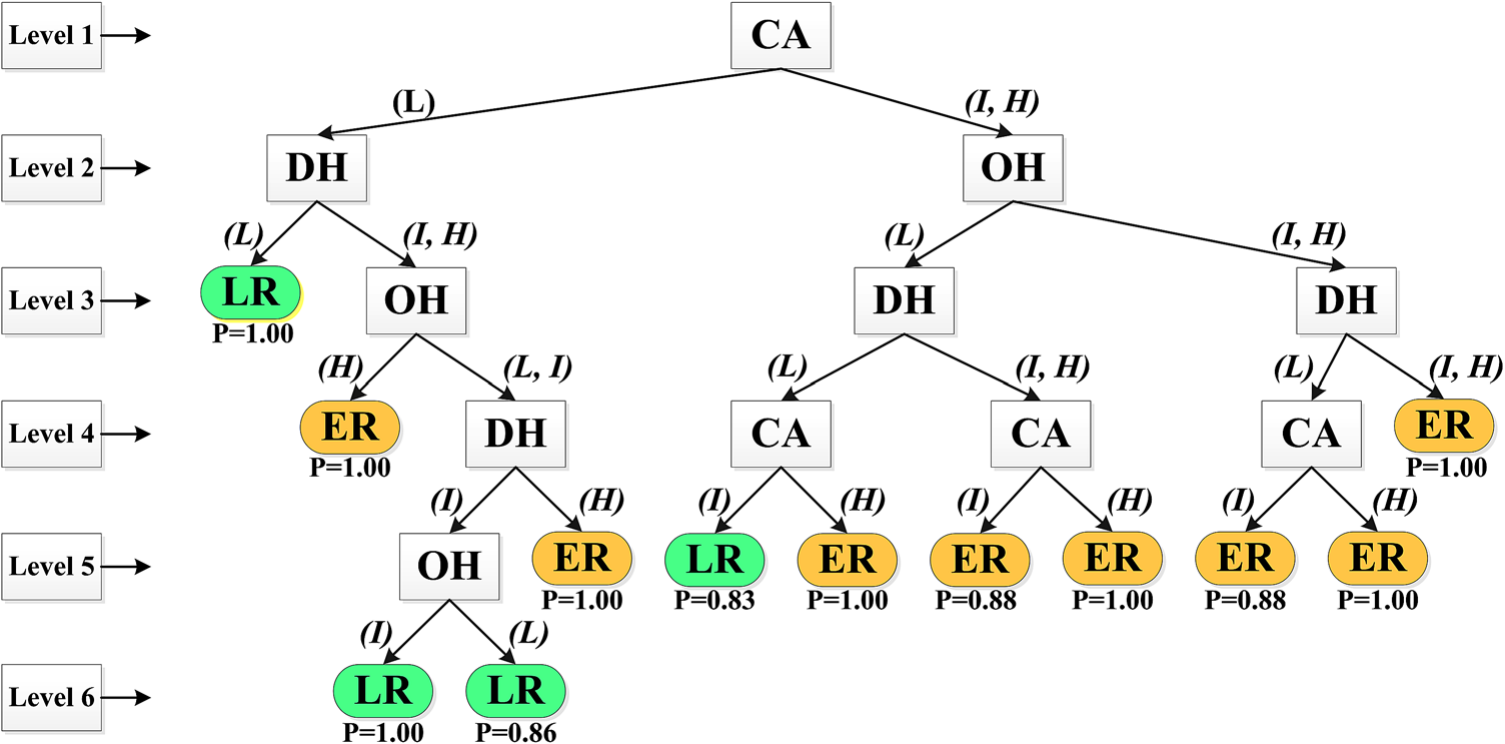
Knowledge tree based on the risk factors “Caries Activity” (CA), “Dietary Habits” (DH), “Oral Hygiene” (OH) and “Medical Risk Factors” from the electronic file system T4. The values for the attributes are “Low risk” (L), “Intermediate risk” (I) and “High risk” (H). As outcomes in the inductive analysis, the caries risk values “Low Risk” (LR) and “Elevated Risk” (ER) were used. The *square boxes* represent an attribute and the *rounded boxes* represent the outcome. In connection with the *arrow*, the value for each attribute is given. Below the outcomes, the probability value (*P*) is shown. Level 1–Level 6 marks the positions in the induced knowledge tree. Values for CA: Low (L) = no or low caries activity; Intermediate (I) = moderate caries activity; High (H) = high caries activity; values for DH: Low (L) = healthy foods; Intermediate (I) = cariogenic diet with moderate intake frequency; High (H) = cariogenic diet with high intake frequency; Values for OH: Low (L) = plaque on few approximal surfaces (PI <20%); Intermediate (I) = general approximal plaque (PI 20–50%); High (H) = more than general approximal plaque (PI >50%.)

In the analysis with the two outcome values “Low Risk” and “Elevated Risk”, the factor “Caries Activity*”* appeared at the top level, thus being the most important factor (Fig. 2). The attribute “Medical Risk Factors” did not appear in the knowledge tree, thus being redundant for the outcome.

The verifying option in XpertRule Analyser showed, in the *training set*, that the outcome value “Low Risk” was correctly classified in 99% and “Elevated Risk” in 93.2%. The correctly classified pattern rules in the *test set* were 99.0 and 93.2%, respectively.

From the results of the inductive analysis, it can be concluded that the pattern rules for the caries risk grouping into “Low Risk” and “Elevated Risk” are realistic.

### Child problems and strengths

Children in the elevated caries risk group had a significantly higher mean value of conduct problems based on the SDQ, compared to those with low caries risk (4.69 vs. 4.15; *p* = 0.041) (Table 3), although the effect size (Cohen’s *d*) was small. No statistically significant differences were found between the low caries risk group and the elevated caries risk group for the other subscales (i.e. hyperactivity–inattention problems, emotional problems, peer problems and prosocial behaviour) (Table 2). For wider comparisons, mean values for parental SDQ from the normative study (Bjornsdotter et al. 2013) are also presented in Table 2.

**Table 2:** Mean values (Mean) and standard deviation (SD) of the low and elevated caries risk groups in relation to the results of the SDQ subscale

SDQ parent	Low caries risk	Elevated caries risk	*t*	*p* value	Cohen’s * d*	Norms^a^
	Mean (SD) (*n* = 153)	Mean (SD) (*n* = 75)				Mean (SD) (*n* = 1361)
Emotion	3.73 (2.44)	4.11 (2.80)	−1.037	n.s.	0.14	1.5 (1.7)
Hyperactiv/Inatt	5.53 (2.57)	6.20 (2.56)	−1.853	n.s.	0.26	2.4 (2.1)
Peer	2.80 (2.16)	2.81 (1.90)	−0.032	n.s.	0.00	1.2 (1.5)
CD	4.15 (1.60)	4.69 (1.97)	−2.070	0.041	0.30	1.1 (1.3)
Prosocial	6.69 (2.14)	6.45 (2.34)	0.748	n.s.	0.12	8.4 (1.7)
Total difficulties	16.22 (5.66)	17.81 (6.31)	−1.928	n.s	0.27	6.2 (4.8)

Norms from the parents of the children aged 10–13 years are presented for comparisons (Bjornsdotter et al. 2013)^a^

*SDQ parent* The Strengths and Difficulties Questionnaire for parents, *Hyper/inatt* hyperactivity–inattention, *CD* conduct disorder; *Peer* peer problems, *Prosocial* prosocial behaviour {generosity and thoughtfulness}, *Total difficulties* all subscales but Pro social behaviour are summed together to a Total Difficulties score, *n* number of children, *p value* level of significance, *Cohen*’*s d* effect size (small = 0.2; medium = 0.5; large = 0.8)

### Disruptive behaviour disorder (DBD)

The mean values from the DBD showed higher mean values for conduct problems and impulsivity in the elevated caries risk group, compared with the lower caries risk group, (0.29 vs. 0.20; *p* = 0.009) and (1.34 vs. 1.10; *p* = 0.021), respectively (Table 3). The effect size (Cohen’s *d*) was between medium and small. For the subscales DBD-Inattention and ODD, no statistically significant differences were found (Table 3).

**Table 3:** Mean values (mean), and standard deviation (SD) from the Disruptive Behaviour Disorder rating scale for parents, Family Warmth and Conflict and Parental Knowledge and Monitoring Scale, for the low versus elevated caries risk groups

	Low caries risk	Elevated caries risk	*t*	*p* value	Cohen’s *d*
	Mean (SD) (*n* = 153)	Mean (SD) (*n* = 75)			
DBD parent					
CD	0.20 (0.17)	0.29 (0.25)	−2.65	0.009	0.42
Inattention	1.38 (0.72)	1.48 (0.82)	−0.94	n.s.	0.13
Impulsivity/hyperactivity	1.10 (0.65)	1.34 (0.78)	−2.34	0.021	0.33
ODD	1.53 (0.61)	1.51 (0.73)	0.22	n.s.	0.03
Family warmth and conflict					
Warmth	19.39 (3.71)	18.56 (4.02)	−1.55	n.s.	0.21
Conflict	9.03 (4.88)	7.11 (5.11)	−2.76	0.006	0.38
Monitoring					
Knowledge	1.76 (0.58)	1.91 (0.61)	−1.71	n.s.	0.25
Disclosure	7.78 (2.76)	7.88 (3.04)	−0.25	n.s.	0.03
Control	1.36 (0.51)	1.49 (0.85)	−1.46	n.s.	0.19
Solicitation	2.26 (0.70)	2.47 (0.77)	−2.01	0.046	0.29
Secrecy	7.88 (1.76)	7.79 (1.88)	0.35	n.s.	0.05

*DBD parent* Disruptive Behaviour Disorder rating scale for parents, *CD* conduct disorder, *ADHD* attention-deficit/hyperactivity disorder here divided in inattention and impulsivity/hyperactivity, *ODD* oppositional defiant disorder, *Knowledge* parental knowledge, *n* number of children, *p value* level of significance, *Cohen’s d* effect size: small = 0.2; medium = 0.5; large = 0.8

### Family Warmth and Family Conflict (FW/FC)

For the Conflict scale, a statistically significant difference was found with a higher mean value in the low caries risk group, compared with the elevated caries risk group for *less conflict* (9.03 vs. 7.11; *p* = 0.006) (Table 3), indicating that there were less conflicts in the families with children belonging to the elevated caries risk group. The effect size was small. No differences were found for warmth in the family.

### Parental Knowledge and Monitoring Scale (PKMS)

Regarding the PKMS, a higher mean value was found in the elevated caries risk group for more parental solicitation (2.47 vs. 2.26; *p* = 0.046) (Table 3), although the effect size was small. For the other subscales, no statistically significant differences were found (Table 3).

### Family structure

The children with an elevated caries risk lived statistically significantly more often in households with more than two children (Table 4). They also had statistically significantly more often a father from a non-Nordic country (Table 4). No relationship was found between the mother’s native country and an elevated caries risk.

**Table 4:** Number of children in the household and the father’s ethnicity in the low and elevated caries risk groups, respectively

	Low caries risk (*n* = 153)	Elevated caries risk (*n* = 75)	Total (*n* = 228)	*p* value	*φ*
Number of children in the household					
1–2 children	109	40	149		
3–6 children	44	35	79	0.008	0.177
Fathers’s ethnicity					
Nordic	107	37	144		
Other countries	46	38	84	0.002	0.201

Effect size is denoted by *φ* (0.5 = strong; 0.3 = intermediate; 0.1 = weak.)

## Discussion

This study has shown that there were statistically significant more children with an elevated caries risk in the study group, compared to children in general, in the Region of Västra Götaland (RVG), both totally and within gender. Differences were found in behavioural characteristics and the family structure in externalising children with an elevated caries risk, compared to externalising children with a low caries risk.

Families from different socioeconomic areas were represented in the study group and their diversity strengthens the study. The high number of mothers answering the questionnaires in this study is worth noting, possibly indicating that mothers are more prone to participate in parental training programmes and take more responsibility when their child has a behaviour problem. It has previously been shown that fathers largely tend to be absent from research and clinical settings related to ADHD, as well as from public forums related to ADHD, such as educational conferences and parental support groups (Singh 2003).

In Sweden, dental care for children, 0–19 years of age, is free of charge and virtually all children attend the regular recall examinations. This makes it possible to collect data from dental records and in the present study, only three children were excluded due to missing records.

The caries risk assessments were made by the examining dentists who were not calibrated specifically for taking part in the present study; however, the R2 risk grouping is self-instructive in the electronic file system. The verification of the pattern rules evolved in the inductive analysis, using the original data forming the R2 risk groups, and pooling the values for intermediate and high caries risk, indicated that the risk grouping used in the present study was relevant.

The internal consistency of the SDQ and the subscales of the DBD are high. The PKMS subscales have been found to be a useful instrument for assessing parental knowledge and parental monitoring with acceptable psychometric properties (Bjornsdotter 2014). The finding that SDQ was significantly correlated with the DBD in the expected direction supports the validity of the SDQ. The available normative data set the results of this study in a wider context, confirming that the study group is characterised by externalising behaviour problems (Bjornsdotter et al. 2013), and that there are significant differences among these children when divided into groups based on caries risk.

The elevated caries risk among children with externalising behaviour problems can to some extent be explained by their behavioural characteristics. Children with more impulsivity and conduct problem behaviour may have difficulties in performing routine activities such as having regular meals and tooth brushing. Good oral hygiene requires persistence, patience and routine, which can be difficult for these children.

Children with externalising problems and an elevated caries risk may share similar temperamental behaviours as children with an ADHD-associated diagnosis, and it could thus be possible to draw parallels with this group of children. It has been found that children with attention-deficit/hyperactivity problems have poorer oral hygiene and an increased consumption of sugary foods (Blomqvist et al. 2011).

Another possible explanation for externalising children having an elevated caries risk may be that these children are challenging in their interactions with parents. Due to the impulsivity and conduct problems, it is possible that rewards with cariogenic treats are sometimes used to manage or distract these children, without considering the consequences for dental health.

A permissive parental attitude to dietary habits and tooth brushing could explain the elevated caries risk, which is in agreement with findings of a Norwegian study (Skeie et al. 2006). The observation of fewer conflicts in families with children with an elevated risk for caries is an interesting finding. The reduced amount of conflicts may be because the family has found a functional approach to the child by avoiding conflicts, using avoidant strategies to balance the child’s temperament to motivate and calm down, or as rewards. Such a pattern can develop over time as a response to the individual needs of an externalising child, with short-term positive consequences for the parents but, which in the long-term, could increase the risk for coercive parenting. If the child’s temperament leads to many conflicts, the parents must choose the most important “fights”, in order to have an acceptable family climate in general. Giving the child more “adult” time, e.g. positive interaction or in other words, increased socialising has been shown to reduce the amount of family conflicts with 50% (Gardner et al. 2006). Therefore, creating positive routines, and setting boundaries would be beneficial.

Since externalising impulsive behaviour may be related to a tendency to develop caries, it is important to identify this group of children at an early stage. Dental treatment of children who are impulsive and act out leads to dental behaviour management problems and in the long run, dental fear. A previous study indicated that externalising children are difficult to treat (Arnrup et al. 2003).

Parents of externalising children with an elevated risk for caries showed more parental solicitation, probably reflecting a concern induced by the behaviour of the child. Children with impulsivity and conduct behaviour problems can have more difficulties following instructions, with the parents worried and concerned about their child. Parental solicitation could be a positive factor for dental care by being more involved in the child’s health promotion and preventive treatment.

The relationship between elevated caries risk and family size is not surprising. The time for each child is restricted in families with more than two children in the household, especially for disruptive children, which leads to limited time for good oral health support. In a previous study of an adult population, it was reported that individuals with low flexibility of daily activities had a lower frequency of tooth cleaning, compared to those who had high flexibility in their daily activities (Abegg et al. 2000).

The elevated caries risk found among disruptive children, who also had non-Nordic father, is in accordance with a previous Swedish study where it was concluded that the parental migration background should be regarded as a caries risk factor (Julihn et al. 2010). Different cultural and ethnic backgrounds, as well as varying oral health and oral care habits, are to be regarded as substantial risk factors for the development of caries.

## Clinical implications

Efforts to identify this group of children and prevent caries are of great value for the child, the parents, and society, since treatment of uncooperative children is time consuming and costly. It is essential that dental care is attentive to impulsivity early on in these children by noting a child’s behavioural characteristics during a dental examination, and to complete an extended history when behaviour is not age appropriate. Based on the SDQ, these could be children who are restless, easily distracted, constantly fidgeting or squirming, and unable to sit calmly in a dentist’s chair. Information regarding a child’s behavioural characteristics can be used by a dentist to modify the caries risk assessment in the software programme R2, and further help clinicians to plan and provide tailored, empirically supported interventions. New ways of reaching these families with information and prophylactic treatment should be developed. Close collaboration between dental care and school health care may develop a successful outcome with no negative experiences.

Since there are parental training programmes available, which have proven to be effective in children with externalising behaviour problems, it would be interesting to know if these programmes affect the oral health behaviour in these children. Parents of children with externalising behaviour problems would probably benefit from an oral health component when participating in parental training programmes. The findings are of clinical relevance and should be considered in therapy planning, dental treatment and prognosis assessment.

## Conclusions

There were statistically significant more children with an elevated caries risk in the study group compared to children in general in the Region of Västra Götaland, both totally and within genders. Differences with regard to behaviour characteristics in externalising children with an elevated caries risk were observed. Children with externalising behaviour and an elevated risk for caries show more impulsivity and conduct problems, compared to externalising children with low caries risk. Furthermore, there were fewer conflicts in the families, but more parental solicitation. A large number of these children lived in households with more than two children and it was more common with a non-Nordic father. Increased knowledge regarding behavioural characteristics in externalising children is an important parameter and should be considered in the caries risk assessment.
